# Preparation of the rainbow trout bone peptides directed by nutritional properties and flavor analyses

**DOI:** 10.1002/fsn3.631

**Published:** 2018-03-26

**Authors:** Weiwei Fan, Xiaoyi Tan, Maolin Tu, Feng Jin, Zhenyu Wang, Cuiping Yu, Libo Qi, Ming Du

**Affiliations:** ^1^ School of Food Science and Technology National Engineering Research Center of Seafood Dalian Polytechnic University Dalian China; ^2^ Shandong Yueyi Biological Technology Co., Ltd Rizhao China

**Keywords:** enzymatic hydrolysis, flavor, nutrition, peptide, protein, rainbow trout

## Abstract

Rainbow trout bone proteins were prepared by heating at 121°C for 30 min, followed by filtration, concentration, and lyophilization. Nutritional properties and flavor analyses of hydrolysates digested by five different enzymes were investigated, respectively. Results showed that the crude protein content of rainbow trout bone was 15.90% and had a well‐balanced nutritional value. The content of total amino acids was 983.64 mg/g. The amount of free amino acids of hydrolysates digested by alkaline protease, neutral protease, flavourzyme, papain, and trypsin for 3 hr was 207.83, 224.13, 1,001.59, 283.26, and 303.64 mg/g, respectively. During the hydrolysis, the main flavor compounds were identified by GC‐MS to be alcohols, aldehydes, ketones, acids, and alkanes. After hydrolysis, the main molecular weight of peptides was focused on the range of 1,000–3,000 Da in all enzymatic hydrolysates. This study provided a theoretical basis to comprehensive utilization of rainbow trout bone in food industry.

## 1 INTRODUCTION

Seafood that contains a variety of essential amino acids, vitamins, and minerals nutritional components is an important food resource to obtain protein in the human diet (Aspevik et al., 2017; Ovissipour et al., 2009). In the process of machining seafood, the main goal is to process products, such as fillets and mince. However, large amounts of protein‐rich residual raw materials will be inevitably produced, such as fish scales, skin, visceral, fish bones, and so on. Fish bones have significant potential for higher value applications in food industry. So a better utilization of this raw material for various applications is a matter of great scientific prospect if they are processed properly.

In recent years, residual raw materials from fish have attracted widespread public concerns, but the limited productivity makes it difficult to take full advantage of them. At present, research on fish bone focused on the following aspects: fish gelatins, bone protein, collagen, chondroitin, and so on (Nagai & Suzuki, 2000). As far as bone protein concerned, many methods such as acidic or alkaline had been tried to extract protein (Arnesen & Gildberg, 2006; ŻElechowska, Sadowska, & Turk, 2010). Compared to the traditional acid/alkali extraction methods, enzymatic hydrolysis technique uses fewer chemicals and costs a shorter extraction time (Yue et al., 2017). Furthermore, enzymatic hydrolysis is an alternative approach to recover biomass from marine species and obtain a soluble hydrolysate that is a more stable, powdered form with a high‐protein content (Guerard, Guimas, & Binet, 2002). On account of the hydrolysis conditions, uncontrolled or prolonged proteolysis usually can generate the highly soluble peptides exhibiting beneficial nutritional properties, but generally lack of the functional properties associated with native protein (Guerard et al., 2002).

The enzyme used in the hydrolysis is a critical factor influencing both the characteristics and composition of hydrolysate and the amino acid sequence of the peptides produced. The types of protease and protein substrate have an effect on the functional properties of the protein hydrolysate. So it is essential to control the process parameters to make it is possible to produce hydrolysate with the desired composition and properties (Pagán, Ibarz, Falguera, & Benítez, 2013).

It was reported that the rainbow trout bone contains 15.9% protein and 17.7% fat (Toppe, Albrektsen, Hope, & Aksnes, 2007). Therefore, this study focused on how to develop a suitable method that obtains the maximum possible recovery of all valuable components from rainbow trout bone. At the same time, assess the nutritional and flavor properties of the protein hydrolysates. Evaluated characteristics contained the degree of hydrolysis (DH), the nitrogen recovery (NR), the molecular weight of the peptides distribution, and main flavor compounds.

## 2 MATERIALS AND METHODS

### 2.1 Materials and chemicals

Rainbow trout bone was supplied by Shandong Yueyi Bio‐Technology Co., Ltd (Rizhao, China). The same batch of rainbow trout bone was stored at a temperature of −30°C and thawed at 0–20°C with a flowing water. Neutral protease (35,000 U/g), alkaline protease (35,000 U/g), flavourzyme (35,000 U/g), trypsin (35,000 U/g), and papain (35,000 U/g) were all purchased from Amresco Co. Ltd (Beijing, China).

### 2.2 Enzyme hydrolysis

Fish bones were cut into a size of blocks of 3–5 cm^3^ approximately. The chopped fish bones were mixed with water at a ratio of 1:1.2 (w/v) and then transferred in extraction pot with a constant temperature of 121 ± 1°C for 30 min. The enzyme was added after the hot‐pressure extraction. The resulting mixture was filtered and centrifuged at 4°C for 20 min at 14,400 g to collect supernatant.

### 2.3 Determination of the degree of hydrolysis

The percentage of free amino groups separated from protein determines the DH that was calculated as the ratio between α‐amino nitrogen and total nitrogen in the samples (Taylor, 1957). The DH was evaluated by the pH‐stat method that was based on the consumed volume of standard NaOH solution to maintain the reaction pH constant, using the following equation described by Adler‐Nissen (Adlernissen, 1986):

(1) $$\text{DH}\;\left(\%\right)=\frac{h-h_{0}}{h_{\mathrm{tot}}}\times 100,$$

where *h* is the content of the ‐NH_2_ group or the –COOH group in the enzymatic hydrolysate, *h*_0_ is the content of the ‐NH_2_ group or the –COOH group in the fish bone, *h*_tot_ is the total number of peptidic bonds in the protein substrate.

### 2.4 Determination of nitrogen recovery

Nitrogen recovery was used as a solubility index of nitrogen to reflect the productivity of the hydrolysis. After the enzymatic hydrolysis, the supernatant was collected by centrifuging at 14,400 g for 20 min. The NR was calculated according to Benkajul and Morrissey (1997):

(2) $$\text{NR}\;\left(\%\right)=\frac{\text{Total nitrogen in supernatant}}{\text{Total nitrogen in substrate}}\times 100.$$

### 2.5 Determination of amino acid composition of hydrolysates

Amino acid identification was performed by high‐performance liquid chromatography (HPLC). Briefly, 0.1 g sample power and 1 ml of 0.1% formic acid‐0.2% methanol‐H_2_O were added into an empty tube, which was then added 4 ml of acidic acetone at −30°C for 24 hr. The resulting mixture was filtered and centrifuged at 4°C for 10 min at 14,400 g to collect supernatant. Afterward, the supernatant was purged with nitrogen to eliminate the organic solvent and redissolved with 0.1% formic acid‐H_2_O.

### 2.6 Determination of volatile compounds of hydrolysates

The volatile compounds in hydrolysates with different enzymes were analyzed by headspace solid‐phase microextraction–gas chromatography mass spectrometry (HS‐SPME‐GC–MS). Two milliliters of hydrolysates was placed into a 10‐ml brown glass vial. In order to make the analyte fully exposed, the SPME devised with polydimethylsiloxane was used for exposing the fiber in the headspace of the vial at 70°C. Take out the fiber, then it was transferred to the gas chromatograph injector port immediately and heated at 260°C for 5 min. The temperature was set as follows: at first, the temperature maintained at 30°C for 3 min, raised to 70°C at a ramp of 2.5°C/min, and to 150°C at 8°C/min. In the end, increased to 260°C at a ramp of 20°C/min and held for 5 min. GC‐MS analysis, using HP‐5 capillary column (5% phenylmethylsiloxane, 30 mm × 0.25 mm, film thickness 0.25 μm; Agilent, USA), was performed on Agilent 7890A. The instrument detector was operated in electron ionization mode with an ionization voltage of 70 eV. Helium was invoked as the carrier gas at a constant flow rate of 1.5 ml/min. The front inlet was kept at 220°C in the splitless mode. Five microliters of 2,4,6‐trimethyl‐pyridine (5 × 10^−4^ mg/ml) was added to hydrolysate as an internal standard.

### 2.7 Determination of molecular weight distribution

Molecular weight distribution was determined by gel permeation chromatography on a Superdex Peptide 10/300GL (GE HealthCare, Sweden). The mobile phase composed of 70% (v/v) acetonitrile and 30% (v/v) distilled water, the flow rate is 0.4 ml/min. Peptides that were known molecular weight (MW) were treated as the standard substance to calibrate the column. Cytochrome *C* (MW = 12,500 Da), aprotinin (MW = 6,512 Da), vitamin B12 (MW = 1,355 Da), glutathione (MW = 307 Da), and glycine (MW = 75 Da) were used as the standards.

### 2.8 Statistical analysis

All data were expressed as the mean ± *SD* performed in triplicates. The analysis of variance (ANOVA) was conducted using SPSS 12 software package (SPSS Thailand Co., Ltd., Bangkok, Thailand). The differences between variables were analyzed by Duncan's new multiple range test. Significant differences were evaluated at *p *<* *0.05.

## 3 RESULTS AND DISCUSSION

### 3.1 Chemical composition analysis

The crude protein content of rainbow trout bone is 15.90%, which was higher than that of milk (3.5%) and egg (13.3%). The fat content is 17.70%, and moisture content of rainbow trout bone is 59.60%. Protein extracted from the rainbow trout bone under the condition of 121 ± 1°C for 30 min, while the extraction rate is up to 80%. Besides, the high content of crude protein and fatty acids proves the rainbow trout bone is apposite to add to food to improve nutritional properties or extract the active ingredient that processed into health products.

The yield of crude protein depends on the method of extraction, raw material used, and the type of deboning machine (Kijowski & Niewiarowicz, 1985). It was reported that 47.5% protein was extracted from minced cod head in dilute NaOH (pH 11) and HCl (pH 2–2.6) (Arnesen & Gildberg, 2006). Another work indicated that 50% soluble protein was available from minced cod backbones while no water added (Lanier, 1995). Whereas a study reported the yield of 11%–17% from total protein content in animal bone (Young, 2010), while our study showed that 80% of the crude protein in the rainbow trout bone can be extracted out using hot‐pressure extraction method. In a word, the method of hot‐pressure process by water can tremendously improve the protein recovery ratio from the bone without using any chemical solvents such as acid or alkali solution and also can avoid the side effects of these solvents.

### 3.2 Comparison of DH and NR by different enzymes

**Figure 1:**
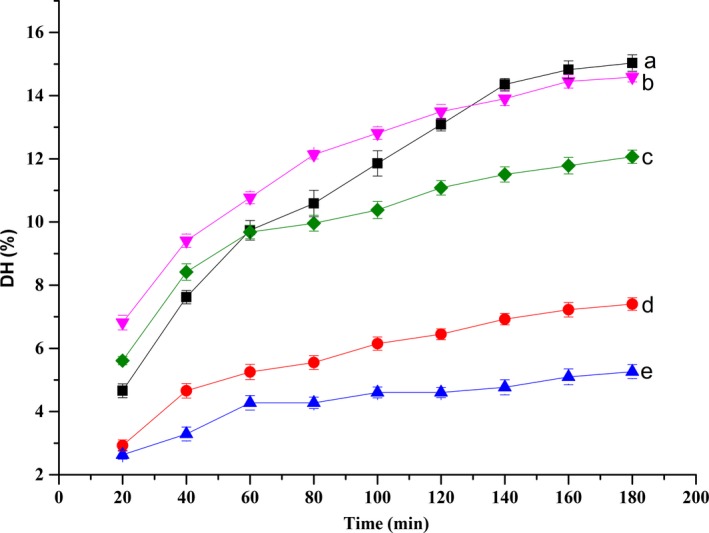
DH of the hydrolysates derived from five different enzyme hydrolysis. The hydrolytic reaction of alkaline protease was carried out at 55°C for 3 hr (pH 8.0), that of papain at 50°C for 3 hr (pH 7.0), that of neutral protease at 50°C for 3 hr (pH 7.0), that of flavourzyme at 50°C for 3 hr (pH 7.5), and that of trypsin at 37°C for 3 hr (pH 8.0). DH, degree of hydrolysis. a: alkaline protease; b: papain; c: neutral protease; d: flavourzyme; e: trypsin

The DH of rainbow trout bone by five different proteases was shown in Figure 1. With regard to the effects of hydrolysis time, it was showed that DH of rainbow trout bone protein treated with alkaline protease, papain, neutral protease, flavourzyme, or trypsin all increased. The highest DH values (15.03%) were achieved with the reaction time of 180 min and hydrolysate treated with alkaline protease (HA) showed apparently higher DH than that treated with other enzymes. At the same time, hydrolysate treated with trypsin (HT) always had the lowest DH. DH was employed as an indicator of the cleavage of peptide bonds. At first, the DH of hydrolysates demonstrated that part of rainbow trout bone extraction was hydrolyzed during hot‐pressure process and decomposed into free amino acids (FAA) and peptides, leading to elevated DH. The curve of DH increased rapidly during the first 2 hr of hydrolysis and then showed a slow increased, which was similar to the results of red hake hydrolysates (Imm & Lee, 1999) and thornback ray gelatin hydrolysates (Lassoued et al., 2015). All peptide bonds susceptible to enzymatic hydrolysis as well as the enzyme inhibition and deactivation could result in the attenuated increasing trend after the starting rise (Ekuwaenyonam, Phillips, & Saalia, 2009).

**Figure 2:**
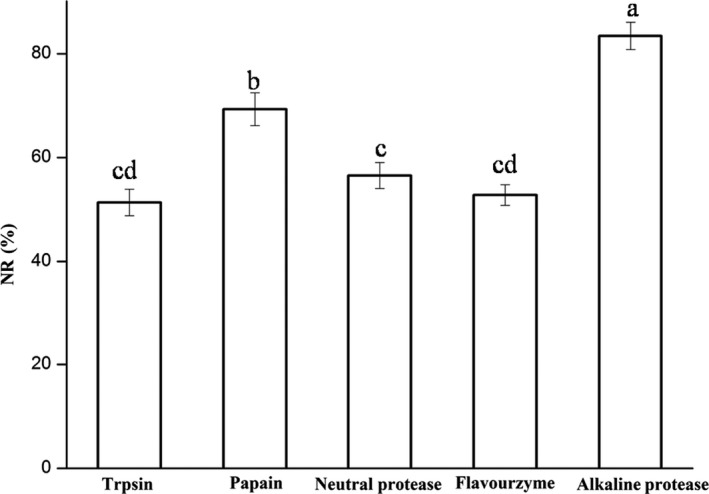
NR of the hydrolysates derived from five different enzyme hydrolysis. The hydrolytic reaction was carried out for 3 hr and the hydrolysates derived from alkaline protease (55°C, pH 8.0), papain (50°C,pH 7.0), neutral protease (50°C, pH 7.0), flavourzyme (50°C, pH 7.5), and trypsin (37°C,pH 8.0), respectively. NR, nitrogen recovery. The values in the same graph followed by different letters are significantly different (*p *<* *0.05)

With the continuation of enzymatic hydrolysis reaction and increase in DH, the NR kept on growing at the same time. NR reflects the yield that can be recovered from the hydrolysis process. Figure 2 showed that NR of rainbow trout bone by five different proteases at the same enzymatic time. The NR value of AH was the highest that reached 83.44%. Changes in DH and NR indicated pretty similar tendency. Similar conclusions were got in tuna waste hydrolysates (Guerard et al., 2002).

### 3.3 Comparison of total amino acids and FAAs of hydrolysates

Total 18 amino acids were well identified from the rainbow trout bone protein. As showed in Table 1, rainbow trout bone protein had a well‐balanced and abundant amino acid composition, the amount of total amino acid was 983 mg/g in rainbow trout bone protein and essential amino acids account for 38.35%. Bitter amino acids account for 43.54% while umami amino acid content was 22.69%. The most abundant amino acid was glutamic acid (132.09 mg/g in rainbow trout bone protein), and the least abundant was tryptophan (3.15 mg/g in rainbow trout bone protein). In general, the enrichment of amino acids will have a positive effect on the diet structure.

**Table 1:** Amino acid compositions of rainbow trout bone protein

Amino acids	Content (mg/g rainbow trout bone protein)
Essential amino acid	
Threonine (Thr)	40
Cysteine (Cys) + Methionine (Met)	38
Lysine (Lys)	72
Phenylalanine (Phe) + Tyrosine (Tyr)	68
Isoleucine (Ile)	32
Leucine (Leu)	63
Histidine (His)	22
Tryptophan (Trp)	3
Valine (Val)	39
Nonessential amino acid	
Serine (Ser)	46
Arginine (Arg)	72
Aspartic acid (Asp)	91
Glycine (Gly)	128
Glutamic acid (Glu)	132
Alanine (Ala)	80
Proline (Pro)	57
Total	983

### 3.4 Volatile compounds in different hydrolysates

Volatile compounds in five hydrolysates for 180 min were identified and were listed in Table 2, which mainly belong to different chemical classes such as alkanes, alkene, alcohols, aldehydes, ketones, pyrazines, ester, and others. The main compounds that play a major role in flavor in hydrolysates are alcohols, aldehydes, and pyrazines. Pyrazines played an important role in the hydrolysates because of their low perception threshold and distinctive characteristic odors (Ryan et al., 2004). Results showed that 71 kinds of volatile compounds contained in the hydrolysate of the rainbow trout bone that treated with alkaline protease. Among them, 30 kinds of hydrocarbons, 20 kinds of alcohols, eight kinds of aldehydes, and eight ketones. Besides, 37 kinds of volatile compounds were identified from the hydrolysate of the rainbow trout bone that treated with papain. Among them, 18 kinds of alcohols, seven kinds of aldehydes, and six ketones. The composition and proportion of the volatile compounds changed with the enzymes system.

**Table 2:** Volatile compounds in the hydrolysates by different enzymes

	RT	Volatile compound	RI	MI	Relative peak area (%)				
					Alkaline protease	Neutral protease	Flavourzyme	Papain	Trypsin
Alkanes	15.209	1‐ethylene‐1‐cyclohexane	893	MS, RI	1.47	0.13	0.07	0.64	0.89
	16.433	N‐(2‐propynyl) pyrrolidine	909	MS, RI	18.90	ND	0.10	ND	ND
	22.239	7‐Propylidene‐bicyclo[4.1.0]heptane	1025	MS, RI	0.79	ND	0.16	ND	1.90
	23.253	4‐methyl‐decane	1051	MS, RI	0.74	ND	0.28	ND	ND
	26.955	1,2‐Epoxy‐undecane	1205	MS, RI	1.43	0.06	0.44	0.99	2.13
	27.123	Dodecane	1214	MS, RI	1.12	0.67	0.22	0.68	1.20
	27.287	4,4‐dipropylheptane	1229	MS, RI	0.71	0.45	0.178	0.99	1.01
	27.935	2‐methyl‐dodecane	1245	MS, RI	0.81	0.64	ND	0.78	0.83
	27.943	6‐ethyl‐undecane	1249	MS, RI	0.88	0.37	0.14	0.56	0.87
	28.011	4‐methyl‐dodecane	1249	MS, RI	0.53	0.06	0.10	2.41	0.57
	28.111	4‐ethyl‐undecane	1249	MS, RI	2.80	0.66	0.35	1.64	2.14
	28.419	4,6‐dimethyl‐dodecane	1285	MS, RI	3.19	0.21	0.15	ND	2.51
	28.572	3,5‐dimethyl‐dodecane	1285	MS, RI	1.21	ND	ND	ND	ND
	28.820	Tridecane	1313	MS, RI	0.82	0.80	0.08	ND	0.48
	28.923	2,6,10‐trimethyl‐dodecane	1320	MS, RI	1.37	0.21	0.19	1.34	1.23
	29.441	6‐methyl‐tridecane	1349	MS, RI	0.44	0.58	ND	0.30	0.30
	30.459	Tetradecane	1413	MS, RI	0.81	ND	ND	ND	ND
	31.256	5,8‐diethyl‐dodecane	1483	MS, RI	0.93	0.46	0.08	0.79	0.69
	31.435	1,1,10‐trimethy‐2‐hydroxyl‐6,9‐epidioxydecalin	1507	MS, RI	0.89	0.90	0.10	0.88	0.65
	31.507	2,6,10‐trimethyl tetradecane	1519	MS, RI	0.66	0.38	ND	1.17	0.52
	31.748	2‐methyl‐Pentadecane	1548	MS, RI	ND	0.18	0.06	0.69	ND
	31.828	3‐methyl‐Pentadecane	1548	MS, RI	0.96	0.07	0.08	0.90	0.68
	31.874	decyl cyclopentane	1555	MS, RI	0.37	0.26	0.06	0.62	0.50
	32.266	5,5‐diethyl‐tridecane	1627	MS, RI	ND	0.17	0.07	0.92	ND
	32.575	3‐Trifluoroacetoxy pentadecane	1650	MS, RI	0.30	0.33	ND	0.42	ND
	32.663	Undecylcyclopentane	1655	MS, RI	0.23	0.43	0.04	0.37	0.30
	32.968	Heptadecane	1711	MS, RI	0.71	0.30	0.08	0.64	0.45
Alkene	15.765	5‐hexenyl‐oxirane	897	MS, RI	0.39	0.51	0.11	ND	ND
	20.825	2,6‐Dimethyl‐2,4,6‐octatriene	993	MS, RI	ND	2.26	ND	ND	ND
	22.308	5‐ethyl‐1‐nonene	1041	MS, RI	ND	2.25	ND	ND	ND
	24.275	5‐t‐butyl‐cycloheptene	1096	MS, RI	3.93	0.53	1.03	8.61	4.69
	25.747	3,3,4‐trimethyl‐1‐decene	1155	MS, RI	1.45	0.43	0.28	1.43	1.19
	30.543	(E)‐1‐tetradecene	1421	MS, RI	1.60	1.05	0.10	0.93	1.27
	30.882	2‐methyl‐Z‐4‐tetradecene	1456	MS, RI	1.88	0.53	0.25	2.27	1.98
Alcohols	9.146	1‐methylcyclopropanemethanol	737	MS, RI	ND	ND	ND	0.53	ND
	10.336	α ‐methyl‐Cyclobutanemethanol	802	MS, RI	3.21	17.43	0.84	10.82	6.41
	16.482	4,5,5‐trimethyl‐tricyclo [2.2.1.0(2,6)] heptan‐3‐ol	917	MS, RI	1.32	0.31	ND	0.52	ND
	19.933	1‐heptanol	976	MS, RI	2.18	ND	0.06	ND	ND
	19.959	4‐sec‐butyl‐2‐butanol‐	964	MS, RI	ND	ND	0.12	0.22	1.04
	20.035	2–heptyne‐1‐ol‐	977	MS, RI	0.64	2.91	0.24	3.03	2.23
	20.329	3‐methyl‐6‐hepten‐1‐ol	985	MS, RI	ND	1.05	0.32	ND	ND
	20.554	(E)‐3‐octen‐2‐ol	987	MS, RI	1.54	ND	ND	ND	0.71
	22.304	4‐methyl‐2‐propyl‐1‐n‐pentanol	1030	MS, RI	6.35	ND	1.70	10.81	7.45
	22.437	3,3‐dimethylcyclohexanol	1042	MS, RI	0.17	ND	ND	1.10	ND
	22.438	Benzyl alcohol	1036	MS, RI	ND	1.40	0.18	0.13	ND
	23.253	5‐methyl‐2‐(1‐methylethyl)‐1‐hexanol	ND	MS	0.13	2.72	ND	ND	1.71
	23.650	(E)‐3‐Nonen‐2‐ol	1086	MS, RI	ND	12.41	ND	ND	ND
	23.669	5‐(methylallyl)‐pentanol	1074	MS, RI	2.54	ND	0.78	7.65	4.36
	24.065	α,α‐dimethyl‐Benzenemethanol	1084	MS, RI	1.92	3.53	0.42	2.24	1.65
	24.107	exo‐2,7,7‐trimethylbicyclo[2.2.1]heptan‐2‐ol	1088	MS, RI	ND	ND	ND	0.59	ND
	25.602	cis‐p‐Mentha‐2.8‐dien‐1‐ol	1140	MS, RI	ND	ND	ND	0.46	ND
	26.177	2‐nonen‐1‐ol	1167	MS, RI	1.28	ND	ND	0.73	ND
	26.685	2‐methyl‐3‐(1‐methylethenyl)‐, (1α,2α,3α)‐cyclohexanol	1196	MS, RI	ND	0.85	ND	ND	ND
	26.776	2‐methyl‐5‐(1‐methylethenyl)‐, (1α,2β,5α)‐cyclohexanol	1196	MS, RI	0.53	ND	0.18	ND	ND
	26.955	(E)‐2‐nonen‐1‐ol	1167	MS, RI	0.28	ND	ND	3.41	0.47
	28.671	2‐methyl‐1‐decanol	1293	MS, RI	0.74	2.26	ND	ND	ND
	29.494	2‐allyl‐1,7,7‐trimethyl‐Bicyclo[2.2.1]heptan‐2‐ol	1350	MS, RI	0.23	2.99	ND	1.91	ND
	29.796	1‐dodecene‐3‐ol	1366	MS, RI	0.86	ND	0.10	0.94	0.80
	31.279	(S)‐2‐methyl‐dodecanol	1492	MS, RI	0.94	0.90	0.12	ND	0.76
	31.916	3,7,11‐trimethyl‐1‐Dodecanol	1563	MS, RI	0.34	0.88	ND	0.81	ND
	32.209	2‐hexyl‐1‐octanol	1591	MS, RI	0.51	0.68	0.07	0.73	0.51
Aldehydes	15.849	Heptaldehyde	905	MS, RI	1.23	7.49	0.44	4.36	2.31
	20.756	2‐ethyl‐2‐hexenal	990	MS, RI	25.25	ND	0.11	0.61	0.64
	21.313	Octanal	1005	MS, RI	ND	ND	0.52	5.11	ND
	24.576	Nonanal	1104	MS, RI	6.44	21.56	1.74	15.94	7.87
	28.671	10‐undecenal	1293	MS, RI	1.17	ND	ND	ND	ND
	29.983	2‐butyl‐2‐Octenal	1379	MS, RI	0.39	1.51	0.10	1.15	1.00
	30.154	2‐butyl‐1‐Octenal	1393	MS, RI	0.41	1.03	0.08	0.78	0.65
	30.406	2‐dodecenal	1410	MS, RI	1.61	1.38	0.13	ND	0.92
	32.000	5‐Chlorobenzaldehyde	1572	MS, RI	2.55	5.20	0.47	3.87	3.21
Ketones	13.897	Tricyclene [4.2.2.0 (2,5)] dec‐7‐en‐3‐one	851	MS, RI	1.72	3.64	ND	2.19	ND
	15.746	2,3‐dimethyl‐cyclopentanone	893	MS, RI	3.29	1.02	1.82	0.13	ND
	19.612	6‐methyl‐3 (2H)‐pyridazinone	975	MS, RI	1.47	ND	ND	ND	ND
	22.765	4‐methyl‐2,4,6‐cycloheptatrien‐1‐one	1047	MS,RI	ND	ND	0.15	1.72	0.19
	26.681	8‐hydroxy‐2‐octanone	1195	MS, RI	0.94	ND	ND	ND	0.53
	27.581	3‐methyl‐3‐decen‐2‐one	1236	MS, RI	ND	0.60	0.73	0.92	ND
	29.830	6,10‐dimethyl‐9‐Undecen‐2‐one	1370	MS, RI	0.12	ND	ND	ND	0.55
	30.917	[1α,2α,4α(E),7α]‐4‐(2,5,5‐trimethyl‐3,8‐dioxatricyclo[5.1.0.0(2,4)]oct‐4‐yl)‐ 3‐buten‐2‐one	1465	MS, RI	0.32	ND	ND	2.13	ND
	31.565	7,8‐dehydro‐8a‐hydroxy‐isophthalate	1523	MS, RI	1.89	2.29	0.36	0.73	ND
Pyrazine	21.156	2‐ethyl‐5‐methyl‐pyrazine	994	MS, RI	13.55	ND	ND	ND	ND
	21.206	2‐ethyl‐6‐methyl‐pyrazine	994	MS, RI	ND	ND	0.46	0.31	3.22
	24.191	2,5‐diethyl‐pyrazine	1093	MS, RI	1.43	0.19	ND	ND	ND
	26.040	3,4‐dihydro‐2H‐benzopyrazine	ND	MS	ND	ND	ND	1.03	ND
Ester	9.123	(Z)‐2‐pentanol acetate	769	MS, RI	ND	ND	ND	ND	1.59
	23.612	Methyl di‐heptane‐2‐carboxylate ester	1069	MS, RI	0.72	ND	ND	ND	ND
	29.102	Cyclohexanecarboxylic acid, 4‐tert‐butyl‐, methyl ester	1322	MS, RI	0.54	0.51	ND	0.51	0.33
	30.985	Benzoic acid, 4‐(chlorocarbonyl)‐, methyl ester	1464	MS, RI	ND	5.63	0.61	4.07	3.65
	31.714	Acetic acid 1,4‐dioxa‐spiro[4.6]undec‐6‐yl ester	1539	MS, RI	0.66	ND	ND	ND	ND
	34.504	Phthalic acid, butyl isoporpyl ester	1873	MS, RI	ND	1.31	ND	0.34	ND
Others	26.040	1,2,4‐trimethyl‐furan	1164	MS, RI	ND	ND	0.12	ND	ND
	30.734	3,7‐dimethyl‐2‐octyl‐1‐alcohol‐isobutyric acid	1437	MS, RI	0.92	1.48	0.11	1.22	0.92

MI, methods of identification; RI, retention indices; MS, mass spectral data; ND, not detect.

The hydrolysate that was treated with alkaline protease contained the most volatile compounds. Among them, the content of 2‐ethyl‐2‐hexenal (25.25%), 2‐ethyl‐5‐methyl‐pyrazine (13.55%), and 4‐methyl‐2‐propyl‐1‐n‐pentanol (6.35%) were relatively high. Moreover, 2‐ethyl‐2‐hexenal is the most important material that causes the fish oil to produce the smell. It is a significant organic intermediate for the production of octanol and spices. Aldehydes have lower thresholds, which were produced by oxidative degradation of polyunsaturated fatty acids in fish oil under the action of enzymes and microorganisms. A class of substances most commonly found in peanut oil is pyrazine, including 2‐ethyl‐5‐methyl‐pyrazine, 2,5‐dimethylpyrazine, and 2‐ethyl‐6‐methyl‐pyrazine. These compounds played a major role in the formation of rainbow trout oil. Ketones make an important contribution to aroma because of its low odor threshold value. Besides, hydrocarbons that produced by the free radicals of fatty acids have little effect on flavor formation because of its high odor threshold.

### 3.5 Distribution of peptides molecular weight

**Figure 3:**
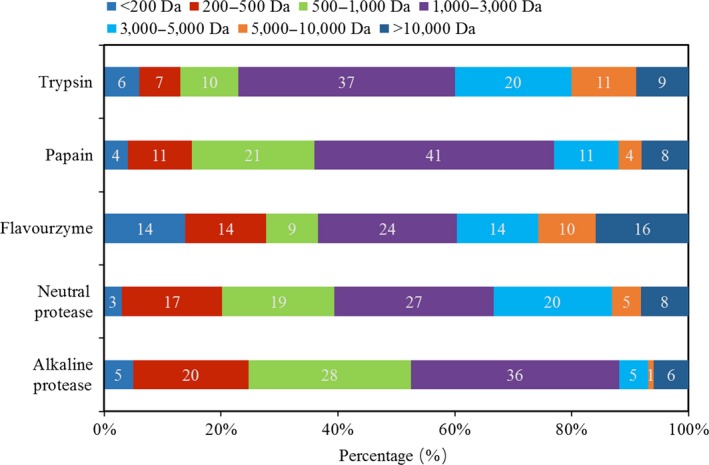
Distribution of molecular weight of peptides in the hydrolysates. The molecular weight distribution was determined by size exclusion chromatography on a Superdex peptide 10/300 GL

The apparent distributions of peptides that are solved by different enzymes were divided into seven ranges and presented in Figure 3. The molecular weight of polypeptides in the five hydrolysates was mainly distributed in the range of 1,000–3,000 Da. The content of small peptides prepared by alkaline protease was the highest, while the content of small peptides hydrolyzed by flavourzyme was the lowest, while the DH of HA was the highest. All these data demonstrated that proteins were degraded into polypeptides and then decomposed into small peptides with the progression of hydrolysis. Similar results were also reported in previous studies (Kristinsson & Rasco, 2000; Ovissipour et al., 2009).

The tendency of the amount of peptides with MW of lower than 500 Da of hydrolysates that digested by different enzymes basically matches the changes in content of FAAs (Table 3). So the components in the fractions with MW lower than 500 Da probably were FAA. Moreover, the highest proportion of the peptides in all of the hydrolysates was the fraction with MW range of 1,000–3,000 Da. Then some peptides were broken down into small peptides, which indicated the potential functional properties and bioactivity of the hydrolysates. Further studies should be investigated to clarify the bioactivity of the peptides.

**Table 3:** Composition and content (mg/g) of FAAs and major taste components of rainbow trout bone hydrolysates powder during the hydrolysis

Amino acids	Concentration (mg/g) (mean ± *SD*)				
	Alkaline protease	Neutral protease	Flavourzyme	Papain	Trypsin
Essential amino acid					
Threonine (Thr)	4.002 ± 0.101^d^	7.381 ± 0.330^b^	44.433 ± 1.612^a^	5.941 ± 0.114^c^	4.706 ± 0.192^cd^
Cysteine (Cys)	0.014 ± 0.001^e^	0.089 ± 0^b^	0.311 ± 0.003^a^	0.049 ± 0.004^c^	0.025 ± 0^d^
Lysine (Lys)	46.483 ± 0.741^d^	42.369 ± 1.322^e^	107.718 ± 1.952^a^	62.001 ± 1.741^c^	92.065 ± 1.806^b^
Methionine (Met)	1.908 ± 0.028^c^	2.117 ± 0.008^c^	32.183 ± 1.577^a^	3.903 ± 0.308^b^	1.297 ± 0.007^c^
Phenylalanine (Phe)	0.306 ± 0.005^c^	0.341 ± 0.001^c^	6.985 ± 0.261^a^	0.605 ± 0.056^b^	0.205 ± 0.001^c^
Isoleucine (Ile)	4.211 ± 0.071^c^	3.526 ± 0.223^d^	8.490 ± 0.171^a^	6.099 ± 0.046^b^	2.756 ± 0.018^e^
Leucine (Leu)	6.476 ± 0.262^c^	5.590 ± 0.232^d^	18.577 ± 0.571^a^	9.415 ± 0.054^b^	4.402 ± 0.022^e^
Histidine (His)	2.546 ± 0.027^d^	6.547 ± 0.068^b^	10.724 ± 0.043^a^	5.424 ± 0.100^c^	2.619 ± 0.038^d^
Tryptophan (Trp)	0.663 ± 0.005^b^	0.895 ± 0.010^b^	4.229 ± 0.776^a^	1.020 ± 0.054^b^	0.668 ± 0.005^b^
Tyrosine (Tyr)	2.814 ± 0.131^c^	3.951 ± 0.094^b^	22.139 ± 1.184^a^	3.406 ± 0.073^bc^	2.950 ± 0.149^bc^
Valine (Val)	8.490 ± 0.078^d^	12.189 ± 0.249^b^	48.639 ± 0.840^a^	9.238 ± 0.140^c^	8.349 ± 0.216^d^
Nonessential amino acid					
Serine (Ser)	7.248 ± 0.088^b^	6.925 ± 0.307^b^	29.154 ± 0.370^a^	6.478 ± 0.182^c^	4.930 ± 0.043^d^
Arginine (Arg)	15.131 ± 0.149^d^	12.209 ± 0.111^e^	74.748 ± 1.852^a^	37.833 ± 1.332^c^	48.304 ± 2.107^b^
Aspartic acid (Asp)	8.250 ± 0.228^c^	12.385 ± 0.753^b^	38.051 ± 0.778^a^	7.669 ± 0.221^c^	6.598 ± 0.024^d^
Glycine (Gly)	12.556 ± 0.716^e^	17.516 ± 0.542^c^	86.979 ± 0.836^a^	27.433 ± 0.516^b^	14.989 ± 0.425^d^
Glutamic acid (Glu)	23.767 ± 0.049^c^	26.999 ± 0.860^b^	74.247 ± 1.875^a^	17.475 ± 0.315^d^	11.145 ± 0.311^e^
Alanine (Ala)	20.005 ± 0.027^b^	20.368 ± 0.593^b^	83.852 ± 1.536^a^	19.331 ± 0.158^b^	13.976 ± 0.101^c^
Proline (Pro)	3.156 ± 0.123^c^	4.303 ± 0.319^b^	7.559 ± 0.128^a^	3.018 ± 0.179^c^	2.862 ± 0.065^c^
Total	207.827	224.130	699.018	283.264	303.637
Major taste component					
Bitter	92.184	94.037	341.991	141.962	166.477
Umami	32.017	39.384	112.298	25.144	17.743
Sweet	43.811	52.190	244.418	59.183	38.601

The values in the same row followed by different letters are significantly different (*p *<* *.05). Bitter: calculated from the sum of leucine, valine, histidine, isoleucine, phenylalanine, methionine, tryptophan, tyrosine, lysine, arginine, and proline; Umami: calculated from the sum of glutamic acid and aspartic acid; Sweet: calculated from the sum of threonine, serine, alanine, and glycine.

## 4 CONCLUSIONS

The present study indicated that alkaline protease was the most efficient enzyme to harvest protein from rainbow trout bone with a relatively higher NR, which was up to 80%. Besides, rainbow trout bone contains high content of protein, which makes it a potential well‐balanced nutritional supplement in various foods. The molecular weight of the main nutritional fractions enriched with peptides compactly distributed at the range of 1,000–3,000 Da. With the increase in DH and NR, 18 kinds of abundant FAA were detected. Volatile compounds test showed the odor change during the hydrolysis process, 71 volatile compounds such as alkanes, alcohols, and aldehydes were totally detected from the hydrolysates.

## CONFLICT OF INTEREST

None declared.
